# Distinctive aspects of the placental epigenome and theories as to how they arise

**DOI:** 10.1007/s00018-022-04568-9

**Published:** 2022-10-26

**Authors:** William A. Pastor, Sin Young Kwon

**Affiliations:** 1 Department of Biochemistry, McGill University, Montreal, QC H3G 1Y6 Canada; 2 Rosalind & Morris Goodman Cancer Institute, McGill University, Montreal, QC H3A 1A3 Canada

**Keywords:** Placenta, Epigenetics, DNA methylation, 5mC, Trophoblast, Development

## Abstract

The placenta has a methylome dramatically unlike that of any somatic cell type. Among other distinctions, it features low global DNA methylation, extensive “partially methylated domains” packed in dense heterochromatin and methylation of hundreds of CpG islands important in somatic development. These features attract interest in part because a substantial fraction of human cancers feature the exact same phenomena, suggesting parallels between epigenome formation in placentation and cancer. Placenta also features an expanded set of imprinted genes, some of which come about by distinctive developmental pathways. Recent discoveries, some from far outside the placental field, shed new light on how the unusual placental epigenetic state may arise. Nonetheless, key questions remain unresolved.

## Introduction

**Fig. 1:**
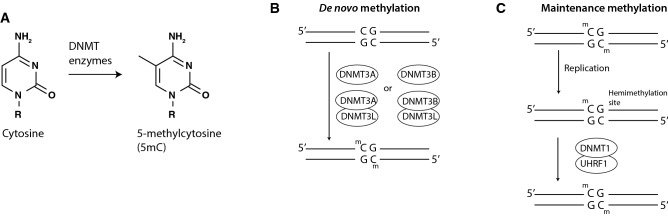
Basics of DNA methylation. **A** Schematic showing methylation of the 5-carbon of cytosine to produce 5-methylcytosine. **B** Illustration of de novo methylation by DNMT3A or DNMT3B, which are sometimes complexed with the catalytically inactive cofactor DNMT3L. **C** Illustration of maintenance methylation. After replication of DNA, the newly synthesized DNA strand is unmethylated forming a “hemimethylation” site in which a methylated CpG is across from an unmethylated CpG. This site is bound by UHRF1 and subsequently methylated by DNMT1

In a wide range of organisms, including all vertebrate animals, the 5-carbon of cytosine can be methylated to form 5-methylcytosine (5mC) (Fig. 1A) [1, 2]. 5mC and its oxidized derivatives are the only epigenetic modifications of the DNA molecule known to exist in animals [3]. The methyl group is exposed on the outward-facing major groove of the DNA double helix and thus affects what proteins can bind to DNA [4]. As such, 5mC can influence chromatin state and modulate transcription [5, 6]. A cell’s genome-wide methylation pattern, or “methylome”, helps shape cellular identity[7]. Correspondingly, different cell types can be identified and distinguished purely on the basis of their “methylomes” [8, 9].

The placenta methylome has attracted interest, both because of its distinctiveness among healthy tissues and because many cancers that arise in somatic tissue recapitulate aspects of the placental methylome [10, 11]. We will first review what is known mechanistically about DNA methylation’s function and how it shapes and is shaped by the chromatin landscape. We will then consider how the placental methylome is distinctive and what is known or can be theorized about how it takes on its unusual aspects. Finally, we will consider what is known about the function of DNA methylation in placental development.

## DNA methylation and chromatin

This topic is reviewed in more depth in other publications[6, 12], but essential details are included below.

### DNA methylation in development

Cytosine is methylated by DNA methyltransferase (DNMT) enzymes. DNMT3A and 3B can impart methylation at previously unmethylated sites[13, 14], a process called “de novo” methylation (Fig. 1B). DNMT3A and 3B can complex with the catalytically inactive DNMT3L, which allosterically increases their enzymatic activity[15]. This methylation typically occurs at cytosines followed by guanine. These sites are termed “CpG sites”, where the p denotes the phosphate that links the two bases [16]. DNMT1 in turn maintains existing patterns of DNA methylation through cycles of cell replication (Fig. 1C), a process called “maintenance” methylation [17]. Maintenance methylation depends on a protein called UHRF1 which recognizes “hemimethylation sites”, a methylated CpG complementary to a newly synthesized and unmethylated CpG, and recruits DNMT1 [18].

The phenomenon of maintenance methylation makes DNA methylation an unusually stable epigenetic mark, but DNA methylation can nonetheless be lost. If DNMT1 fails to act with perfect efficiency, 5mC will be diluted out through cycles of cell division [5]. Tet enzymes in turn can oxidize 5mC to 5-hydroxymethylcytosine (5hmC) [19], which is less efficiently recognized by DNMT1 [20, 21]. *TET* enzymes can further oxidize 5mC to 5-formylcytosine and 5-carboxycytosine, which are excised by the glycosylase TDG and replaced by cytosine in the course of base excision repair [22, 23]. Replication-dependent and -independent 5mC loss are called “passive” and “active” demethylation, respectively [5].

**Fig. 2:**
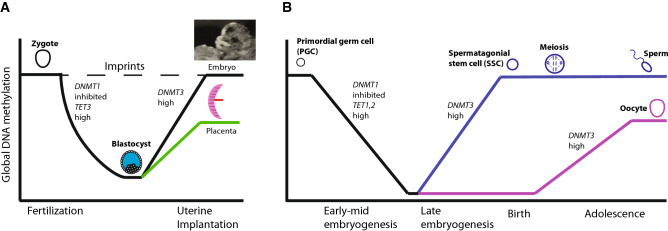
DNA methylation dynamics during development. **A** Methylation dynamics during early mammalian embryogenesis. **B** Methylation dynamics in germline during late embryogenesis and subsequent development. Note that the sperm and egg develop substantially different methylomes during this period, setting the basis for imprinting in the next generation

A mass reprogramming of DNA methylation occurs during the first few days of mammalian embryonic development. A few hours after fertilization, before the pronuclei from the sperm and egg have fused, the paternally inherited DNA undergoes near total demethylation (Fig. 2A) [24]. It is at present unclear whether this is a TET-mediated process [25–27]. DNA methylation is then lost passively from the maternally inherited DNA, and by the blastocyst stage, global DNA methylation levels are low [28–30]. Subsequently, DNMT3A and 3B are upregulated and genome-wide de novo DNA methylation occurs [28]. By this point, the epiblast, trophoblast and primitive endoderm lineages have already been specified, and they acquire disparate patterns of DNA methylation [31–33].

Not all DNA methylation is lost during pre-implantation reprogramming; the blastocyst never reaches zero methylation in mice or in humans [29, 30]. Notably, a handful of imprinting control regions (ICRs) retain methylation inherited from the parental gametes. These ICRs are methylated in the sperm or oocyte but not both, and retain selective methylation in the paternally or maternally inherited copy throughout pre-implantation reprogramming and during subsequent embryonic development. Accordingly, they impart selective expression of paternal or maternal alleles of nearby genes (discussed below and reviewed in [34, 35]). However, the vast majority of the genome is effectively reset and re-established in early embryogenesis. Subsequent methylation changes in placental and somatic development are relatively modest by comparison [36], although germ cells undergo a second global demethylation/remethylation event (Fig. 2B) [37].

### How DNA methylation shapes chromatin

DNA methylation typically antagonizes transcriptional initiation and thus silences gene expression [38, 39]. It does so via several mechanisms. A number of widely expressed transcription factors have recognition motifs that contain CpG sites and have reduced binding to 5mC [40–42]. Several proteins, including proteins that mediate H3K4 methylation, H3K36 demethylation and Mediator recruitment, contain CXXC domains that specifically bind to unmethylated CpG [43]. Finally, there exist proteins (MBD1, MBD2, MBD4, MeCP2 and Kaiso) that specifically recognize 5mC and recruit histone deacetylases and H3K9 methyltransferases [44–47]. 5mC thus promotes a heterochromatic state of deacetylated, H3K9-methylated chromatin. It should be noted that some of these silencing mechanisms show strong “density dependence”: a single 5mC will not have a major effect, but a cluster of 5mC in a small stretch of genome can induce heterochromatinization [39, 45].

### How chromatin shapes DNA methylation

**Fig. 3:**
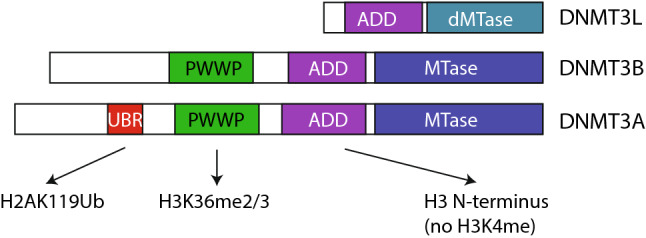
Domain structure of mammalian de novo methyltransferases. DNMT3A and 3B have an active methyltransferase (MTase) domain, while DNMT3L’s MTase domain is inactive. All three DNMTs have an ADD domain that binds to H3 N-terminus and is antagonized by H3K4 methylation. DNMT3A and 3B have a PWWP domain that recognizes H3K36 methylation, and the longest isoform of DNMT3A has a ubiquitin binding region (UBR) which recognizes H2AK119ub

The de novo DNA methyltransferases lack strong sequence preference, but their activity is affected by underlying chromatin modifications (Fig. 3). All DNMT3 proteins have an ADD domain which binds to the N-terminus of histone 3 tail, [48] and methylation of lysine 4 strongly inhibits ADD domain binding [49–52]. Since H3K4 methylation is typically found at promoters and enhancers, this “protects” these sites from DNA methylation during the period of de novo DNA methylation. DNMT3A and 3B also have PWWP domains that target them to H3K36 methylation sites, which are found in gene bodies (downstream of the promoter) of transcribed genes and some intergenic regions [52–55]. Finally, DNMT3A isoform 1 contains a ubiquitin binding region which recognizes the modification H2AK119ub, a histone mark imparted by Polycomb Repressor Complex 1 (PRC1) [56].

A critical implication of these targeting mechanisms is that cells with different underlying transcriptional patterns, such as early embryonic lineages, will acquire DNA methylation differently.

### The phenomenon of CpG islands

Because 5mC is prone to undergo C to T transition mutations, heavily methylated genomes mutate CpG to TpG over evolutionary time [57]. As a result, the vast majority of the genome has a far lower density of CpG sites than would be mathematically expected from the abundance of C and G bases [58]. However, there exist thousands of “CpG islands” which have both an elevated GC content and a CpG content close to what is mathematically expected [58, 59]. Most CpG islands are never methylated, explaining how they can retain CpG across evolutionary time.

These CpG islands are of critical importance for mammalian gene regulation. As discussed above, a variety of transcription factors and CXXC domain containing proteins bind CpG sequences. CpG islands are thus hubs of transcription: more than half of all mammalian promoters are CpG islands, including virtually all constitutively expressed genes [58]. When CpG islands do undergo methylation, the 5mC density is very high and heterochromatinization and silencing are correspondingly effective [45].

## Features and acquisition of the placental methylome

**Fig. 4:**
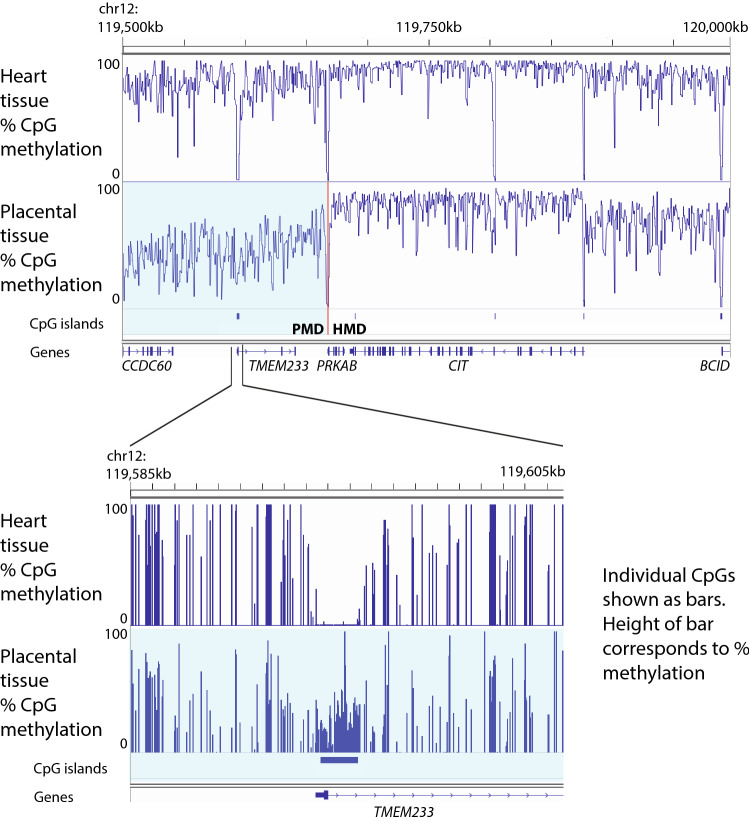
Pattern of DNA methylation in human soma and placenta. A 500 kb region of chromosome 12 is shown to illustrate DNA methylation patterns in soma and placenta. DNA methylation is generally high in soma with dips at regulatory elements, such as gene promoters, CpG islands and enhancers. In placenta by contrast a large partially methylated domain (PMD) spanning hundreds of thousands of bases is present, and is shaded in light blue. Shown in inset is an example of CpG island which is unmethylated in soma but has substantial methylation in placenta. The CpG island in question is the promoter of the gene *TMEM233*, which is expressed in certain somatic lineages but not placenta. Data are from ENCODE libraries generated by the Bradley Bernstein lab (ENCSR739XWV, ENCSR699ETV) [147, 148]

Somatic cells in mammals have a methylome with the following properties: (1) low methylation at active promoters, (2) low methylation at most CpG island promoters (including those inactive in the cell type in question), (3) intermediate methylation at enhancer elements and (4) high methylation elsewhere in the genome. In other words, 5mC is essentially universal except at regulatory elements a few hundred to a few thousand bases wide (Fig. 4).

The placental methylome differs from its somatic counterpart in several dramatic ways (Fig. 4). It has a much lower global level of 5mC. Low 5mC is a common feature of eutherian placentas and is also observed in the extraembryonic membrane of marsupials [60]. The placenta is also unusual in containing large regions of genome with intermediate levels of DNA methylation. In some species, including humans, the placental epigenome contains “partially methylated domains” (PMDs), regions of hundreds of thousands or millions of bases with intermediate levels of DNA methylation, interspersed with “highly methylated domains” (HMDs) where methylation is high except at promoters and enhancers [11]. In other species, such as mouse, the distinction between PMDs and HMDs is more subtle, but the feature of widespread intermediate methylation is retained [60, 61]. The placental methylome is also distinct insofar as there are hundreds of CpG islands, including promoters of many genes important for somatic development, which are specifically methylated in placenta [11, 33, 62, 63] (Fig. 4). Finally, the placental methylome contains an expanded set of imprinted loci, some of which come about via unique mechanisms [35]. Each of these phenomena will be considered in turn.

### Low global DNA methylation and the start of partially methylated domains

**Fig. 5:**
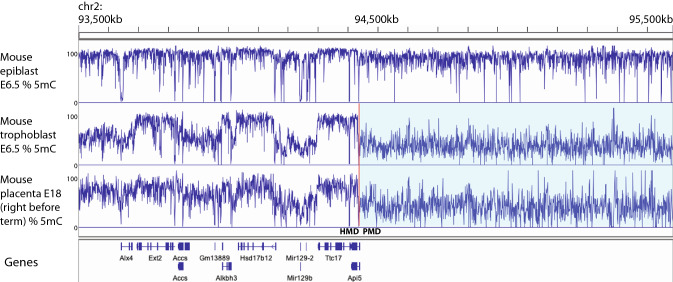
Methylation dynamics during mouse development. DNA methylation in E6.5 mouse epiblast, E6.5 mouse trophoblast and E18 mouse placenta. A rolling average of %CpG methylation over the region is indicated. Note that by E6.5, DNA methylation is generally high in epiblast but much lower in trophoblast. In E6.5 trophoblast, high DNA methylation is apparent over transcribed gene bodies and low elsewhere. By E18, this pattern is less striking, but a region with several expressed genes now forms a broader HMD, while the region without expressed genes is a PMD (shaded in light blue). Data are from published sources [61, 64]

During the wave of DNA methylation that occurs during the peri-implantation period, the epiblast undergoes far more extensive DNA methylation than the trophoblast [31, 33, 64]. Notably, whereas virtually all the genome except regulatory elements is heavily methylated in epiblast, large regions of partial methylation remain in the trophoblast (Fig. 5). PMDs form initially simply because some regions are never highly methylated in trophoblast.

Here it should be noted the general pattern in all genome-wide de novo DNA methylation events is that transcribed gene bodies and nearby regions are methylated first, with transcriptionally inert regions showing slower DNA methylation [31, 32, 52]. In epiblast and male germline, eventually almost the entire genome is methylated. In trophoblast, primitive endoderm and oocyte, large regions of the genome remain incompletely methylated. The reason for this difference is unclear: it may be that epiblast and male germline have such high levels of DNMT3 activity that methylation activity eventually “saturates”, while this does not occur in the other cell types [32]. Alternatively, differences in underlying chromatin distribution may be critical. It is noteworthy that divergent patterns of H3K36 methylation  are predictive of the differences in DNA methylation patterning in male and female germline [12, 65, 66]. Regardless, partially methylated domains arise in placenta because methylation of transcriptionally inert regions is incomplete (Fig. 5).

### Perpetuation and further methylation loss over partially methylated domains

**Fig. 6:**
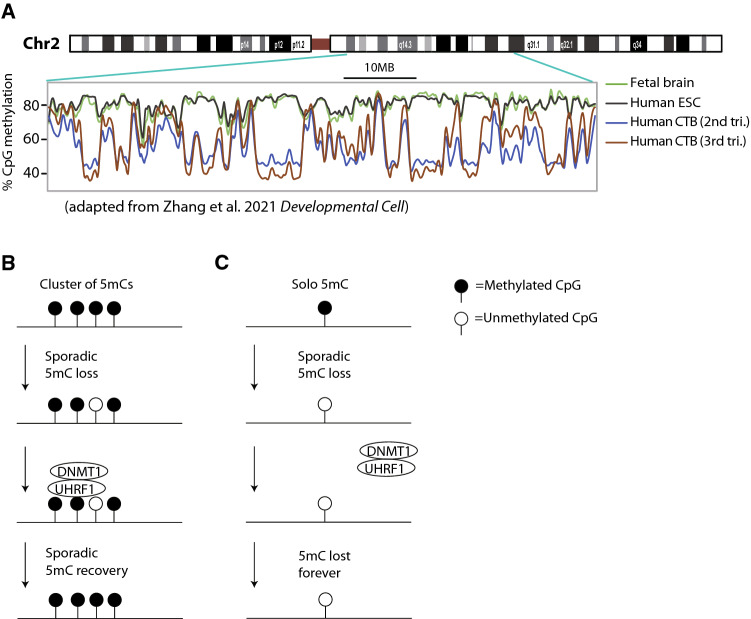
Erosion of DNA methylation over PMDs. **A** DNA methylation over a ten million base region of chromosome 2. Brain and embryonic stem cells (ESCs) show uniformly high DNA methylation. Cytotrophoblasts (CTBs) show lower global methylation with distinct PMDs. Note that in CTBs, DNA methylation is gained in HMDs but lost in PMDs between the second and third trimesters. **B, C** Proposed mechanism of PMD erosion. In a region with several methylated CpGs in close proximity, sporadic loss of a single 5mC can be reversed by DNMT1 homing to nearby 5mCs (**B**). Methylation erosion at an isolated 5mC, however, cannot be reversed (**C**)

After the peri-implantation wave, DNA methylation in the trophoblast is not completely static. Intriguingly, while DNA methylation as whole increases between the second and third trimester, DNA methylation in PMDs drops further, solidifying the distinction between HMDs and PMDs [67] (Fig. 6A). A similar trend is observed in the short course of mouse gestation [61]. Cultured human trophoblast stem cells (hTSCs) derived from first trimester cytotrophoblasts show this phenomenon to an extreme degree, exhibiting near-complete loss of 5mC over PMDs [68]. To understand this 5mC erosion, studies conducted in other tissues are highly relevant.

In addition to placenta, PMDs have been frequently detected in cancers and cultured cells and exist in a more subtle form in healthy tissues [69, 70]. The strong general trend is that AT-rich, CpG-poor, transcriptionally inactive regions are prone to become PMDs. Note that while base composition does not vary between cell types, transcriptional activity does, and as such there is only partial overlap of PMDs between different cell types [70]. Erosion of 5mC over PMDs is observed over the normal course of human aging and is accelerated in fast-dividing cancers [71]. Within PMDs, isolated CpGs lose DNA methylation faster than clusters of CpGs [69].

Zhou and colleagues demonstrate a plausible model to explain all these findings. The central idea is that somatic/cancer PMDs can arise by incomplete DNA methylation maintenance across cell replication cycles [69]. Transcriptionally inert regions are late replicating [72], so there is less time for DNMT1 to act before mitosis and a higher chance of a CpG being “missed” [73]. Furthermore, isolated CpGs are especially prone to lose methylation. DNMT1’s activity can best be understood as zonal rather than precise: in a region with methylated CpGs it will impart more methylated CpGs after replication, but not necessarily at the exact same CpGs [74, 75]. As such, a cluster of methylated CpGs in close proximity can resist demethylation: even if DNMT1 misses one CpG in a given replication cycle, DNMT1 will still bind the region and potentially remethylate the CpG in later cycles (Fig. 6B). If an isolated CpG loses methylation by contrast, 5mC is lost forever because DNMT1 will no longer bind nearby (Fig. 6C). Hence, AT-rich, CpG-poor regions lose 5mC slowly over the course of normal human aging, with accelerated loss in rapidly dividing cancers and cultured cells [69].

The Zhou model can be adapted to explain PMD formation in placenta. The placenta starts out with less DNA methylation over large swathes of the genome than somatic tissue, particularly over less transcribed DNA. The isolated methylated CpGs that do exist in PMDs are especially prone to loss in subsequent replication cycles because there is less 5mC around them. This results in further 5mC erosion in PMDs over the course of pregnancy and dramatic 5mC erosion for rapidly-dividing hTSCs in culture.

### The chromatin state of PMDs

Placental PMDs show striking enrichment of H3K9me3, and some enrichment for H3K27me3 is also observed [67]. This finding may appear incongruous, because regions of dense DNA methylation are known to attract H3K9me3 as described above. However, it is well established that H3K9me3 establishment can occur independently of 5mC. Indeed, H3K9me3 is present in organisms that lack 5mC (e.g. yeast, nematodes, *drosophila*) and mechanisms for 5mC-independent H3K9me3 establishment are known in mammals, such as the KAP1 complex [76] and HUSH complex [77].

**Fig. 7:**
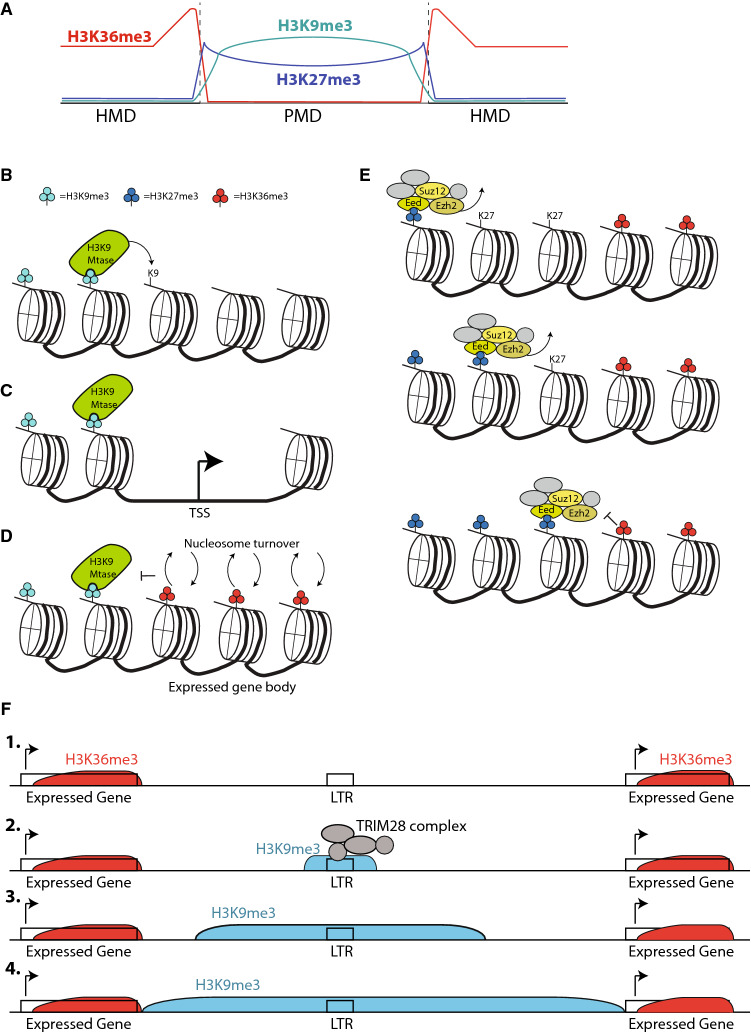
Spread and containment of heterochromatin in PMDs. **A** Pattern of heterochromatin marks in PMDs, based on literature in cancer. H3K9me3 is enriched at the heart of PMDs, H3K27me3 is enriched closer to PMD/HMD boundaries and H3K36me3 is absent from PMDs but enriched at the HMD side of the boundary, reflecting transcription’s role as a barrier to heterochromatin spread. **B** Because some H3K9 methyltransferases can recognize H3K9me3, either directly or via interactor proteins, H3K9 MTases can bind H3K9me3 at one nucleosome and methylate H3K9me3 on adjacent nucleosomes, thus spreading heterochromatin. **C** Regions of nucleosome-free DNA can block the spread of heterochromatin. **D** Rapid turnover over nucleosomes over transcribed gene bodies can block the spread of heterochromatin. **E** H3K36me3 blocks binding of EED and spread of H3K27me3. **F** An example of how TRIM28 complex-mediated H3K9 methylation could potentially seed the formation of a large region of heterochromatin

Likewise, there is extensive precedent in cancer for H3K9me3 and H3K27me3 enrichment over PMDs [78]. These heterochromatin marks are sometimes found together, though H3K9me3 is more enriched in larger PMDs, while H3K27me3 is more enriched at smaller PMDs or near the edges of large PMDs [70, 78, 79]. H3K36me3, associated with active transcription, is enriched immediately outside the PMDs, essentially forming the HMD/PMD boundaries (Fig. 7A) [70]. Expressed genes are typically found on the HMD side of HMD/PMD boundaries in placenta as well [60].

How could this chromatin state form and how could it give rise to PMDs? The formation of large blocks of heterochromatin during development is a widespread phenomenon. While embryonic stem cells show heterochromatin at discrete foci, differentiated cells show large regions of H3K9me2, H3K9me3 and H3K27me3 [80–82]. This developmental transition likely reflects the inherent propensity of heterochromatin to spread [83]. The SUV39 H3K9 methyltransferases themselves recognize H3K9 methylation and can thus bind H3K9me3 on one nucleosome and trimethylate H3K9 on the next (Fig. 7B). Likewise, the PRC2 component EED recognizes and is stimulated by H3K27me3 [84, 85]. While it is somewhat unclear the extent to which these marks are spread by two- vs. three-dimensional diffusion, they have a clear capability to spread until they hit a barrier. Active transcription can function as such a barrier, and can block the spread of heterochromatin in several ways. Nucleosome turnover associated with transcription has the effect of blocking the spread of heterochromatin, as do the nucleosome-free regions found at active gene transcription start sites [86] (Fig. 7C, D). Finally, H3K36 methylation directly inhibits PRC2 [87, 88] (Fig. 7E).

A combined model of placental PMD formation runs as follows. Any one of a number of silencing pathways seeds heterochromatin formation at a given place in the genome. For example, the TRIM28 complex acts very early in embryonic development, creating patches of H3K9me3 before and during specification of trophoblast [89]. To cite another known example, the transcript *Airn* recruits PRC2 and the H3K9 methyltransferase G9a, creating an 10 Mb block of heterochromatin [90]. Heterochromatin spreads from the seeding site until it reaches an insulator or actively transcribed gene (Fig. 7F). This produces the pattern observed at PMDs in which H3K9me3 and/or H3K27me3 are enriched right up to a patch of H3K36me3 (Fig. 7A) corresponding to transcribed genes. Because their mechanism of formation precludes inclusion of active genes, on average these heterochromatin blocks will contain fewer CpG islands and exons (which are also CpG rich) and thus be generally CpG poor. Being heterochromatin, they replicate late in cell cycle and thus have less efficient maintenance methylation. Thus, these heterochromatic blocks become the PMDs we see in placenta. Bounded by H3K36me3, which attracts de novo DNA methylation, sharp boundaries between regions of high and low DNA methylation become apparent.

One factor potentially tempering the loss of DNA methylation over PMDs is the ability of the UHRF1 tandem Tudor  domain to bind H3K9me2/me3, thus helping recruit DNMT1 to heterochromatic regions [91, 92]. Interestingly, one report indicates reduced overall maintenance methylation efficiency upon trophoblast giant cell differentiation [93], and it is unclear if there are broader differences in maintenance methylation efficiency in trophoblast as compared with other lineages.

Might PMDs in turn attract heterochromatin? There is evidence that 5mC antagonizes PRC2 activity [94], so methylation loss could potentially promote H3K27me3 acquisition. Indeed, metabolic changes caused by acquisition of drug resistance in breast cancer feature rapid hypomethylation and H3K27 methylation acquisition [95]. Analysis of liver shows PMDs expanding, and H3K9me3 enrichment increasing, in increasingly cancerous cell lines [70]. Hence, self-reinforcing cycle may target heterochromatin to PMDs and promote DNA methylation loss over heterochromatin.

### Placenta-specific CpG island methylation

The origins of placenta-specific CpG island methylation are at best partially understood. Deletion of the H3K27 methyltransferase *Ezh2* results in hypomethylation of these regions in the extraembryonic ectoderm (future placenta) of mice [33]. This finding in turn raises questions we do not at present have answers to. How does EZH2, or its resultant histone mark H3K27me3, promote 5mC acquisition? As discussed above, the de novo DNA methyltransferase DNMT3B has the ability to bind to H3K36me3 and DNMT3A can bind to H3K36me2, H3K36me3 and H2AK119ub [56], but no direct interaction of a DNMT with H3K27me3 has been demonstrated. There is crosstalk between the PRC2 and PRC1 pathways, such that loss of *Ezh2* could result in perturbation of H2AK119ub [96]. However, ablation of *Rnf2*, the PRC1 component which catalyses H2AK119 ubiquitination, does not result in hypomethylation of CpG islands extraembryonic ectoderm [64]. Furthermore, DNMT3B, which does not bind to H2AK119ub, is primarily responsible for DNA methylation in trophoblast [33]. An alternative possibility is that the PRC2 complex itself recruits DNMTs. Indeed, all four DNMTs have reported physical interactions with PRC2 components [97–99]. A final possibility is that EZH2 is required for silencing of transcription and concomitant DNA methylation. As discussed above, H3K4 methylation antagonizes DNMT3 binding and activation [49, 50]. Hence, it is possible that EZH2 represses the target genes, which will have the effect of preventing H3K4 methylation and promoting DNA methylation. However, neither of these plausible mechanisms have been demonstrated to be correct. Equally unclear is why EZH2/H3K27me3 attracts methylation specifically in the trophoblast lineage, given that this protein and mark are present at the exact same loci in the developing epiblast [100].

It is worth noting that aberrant CpG island methylation in regions of H3K27me3 is an extremely common phenomenon in cancer, potentially important in the silencing of tumour suppressors [101]. The mystery of why H3K27me3 attracts DNA methylation in trophoblast is thus of both scientific and medical importance.

### Imprints

**Fig. 8:**
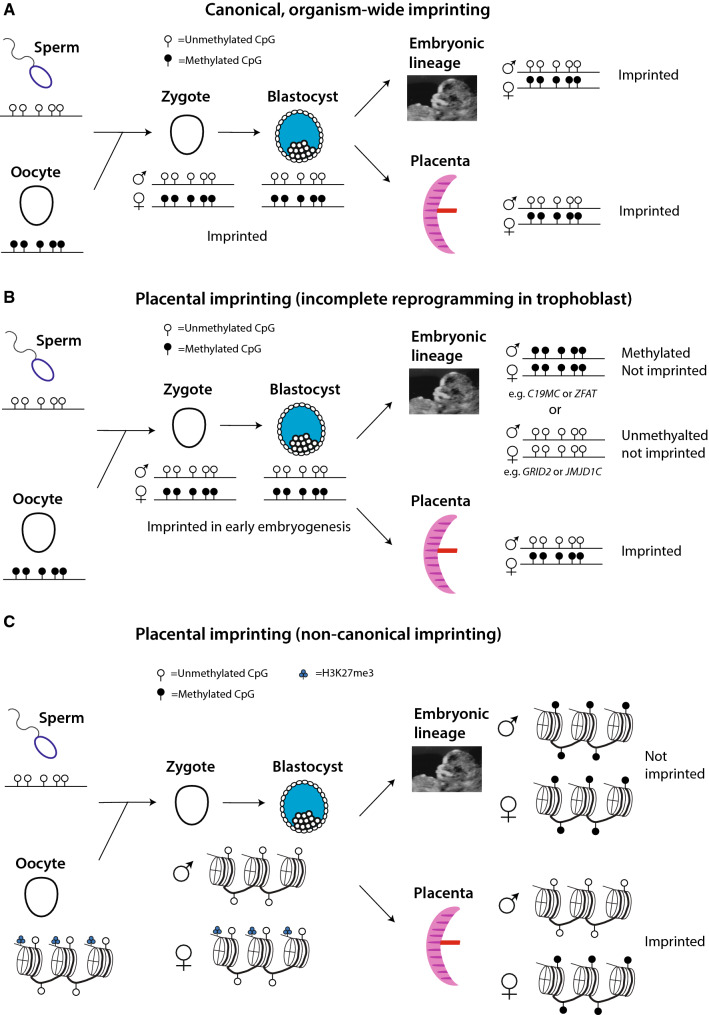
Mechanisms of imprinting. **A** Example of canonical, organism-wide imprinting. A region of the genome is methylated in sperm or oocyte, but not both, and this differential pattern of methylation is preserved through early embryonic development and in the embryonic and placental lineages. **B** Example of how placental imprinting can arise through incomplete reprogramming in trophoblast. The locus is methylated in oocyte but not sperm and retains this differential methylation pattern through pre-implantation development. In the epiblast lineage, either the paternal copy is methylated or the maternal copy is demethylated, and the locus is not imprinted. In the trophoblast lineage, the locus retains parent-of-origin-specific methylation. **C** Non-canonical imprinting. Methylation is not inherited from parental gametes (or is rapidly lost after fertilization). Instead, H3K27me3 is inherited from the oocyte and the maternal copy is methylated after implantation. Typically in the epiblast lineage, both copies of the locus are methylated

Imprinted genes have the distinct characteristic that only one allele (paternal or maternal) is expressed. Canonical imprinting entails parent-of-origin-specific methylation of an ICR which controls expression of a nearby gene or cluster of genes (Fig. 8A). In some cases, regulation of an imprinted gene is relatively simple: a gene’s promoter is methylated in the allele inherited from one parent and the other allele is expressed [102]. Other loci have more complex modes of regulation, in which methylation of an ICR directly represses one gene but activates other genes via indirect mechanisms, for example, by silencing a transcript that silences other transcripts in cis [103] or by blocking binding of the insulator CTCF [104].

As a mechanism of gene regulation, imprinting is evolutionarily disadvantageous to the extent that it sacrifices the advantage of a biallelic genome: having two expressed alleles of a gene hedges against the danger of mutation at one allele. Presumably there is some compensatory evolutionary advantage to imprinting. Imprinting clearly impedes parthenogenesis, an unfertilized oocyte giving rise to a pregnancy, because embryos with maternal-only imprinting cannot develop [105, 106]. One function of imprinting may therefore be to block parthenogenesis. A related theory proposes that imprinting prevents oocytes from spontaneously giving rise to aggressive ovarian teratomas or trophoblastic tumours, because trophoblastic development requires male imprinting [107]. Perhaps the best accepted theory postulates that imprinting is the product of an evolutionary “battle of the sexes”: it is in the father’s interest to maximize maternal energy investment in his progeny, whereas it is in the mother’s interest to retain energy for future pregnancies [108]. In support of this model, loss of maternal imprints typically produces overgrowth, while biallelic maternal imprinting produces smaller progeny [109, 110].

Regardless of telos, imprinting seems to be especially important in placenta. Some of the most highly expressed genes in the placenta are imprinted, and imprinting defects produce striking placental defects, including the phenomenon of molar pregnancy in humans (discussed further below). Furthermore, in both mice and humans, there are a substantial number of genes that are only imprinted in placenta; in somatic tissues these genes are either expressed biallelically or not at all [111–116].

Placental-specific imprinting may arise in a number of ways. One mechanism is reprogramming of parental methylation in epiblast but not trophoblast. A large number of loci are “transiently imprinted” in early development. Because the male pronucleus is demethylated more rapidly and completely than the female pronucleus, from the 1-cell to blastocyst stage, large swathes of the genome selectively retain maternal methylation [30, 117, 118]. During the peri-implantation stage, methylation is either gained at the paternal allele or lost at the maternal allele, ending the locus’ brief period of imprinting [117]. A number of such loci retain selective maternal methylation in the trophoblast but not somatic lineage. At some such loci (e.g. *ZFAT*, *C19MC),* the paternal allele is methylated in somatic lineage (Fig. 8B). At other loci (e.g. *GRID2, JMJD1C*), the maternal copy is demethylated in somatic lineage [115]. The common feature is that reprogramming of the parental methylome is less complete in trophoblast lineage, giving rise to placenta-specific imprinting.

Alternatively, “non-canonical” imprinting mechanisms can result in placenta-specific imprinting [114]. The key feature of non-canonical imprinting is that DNA methylation is not inherited from the parental gametes. At some non-canonically imprinted loci, H3K27me3 rather than 5mC is inherited from the mother. These imprints only acquire 5mC upon implantation [119, 120] (Fig. 8C). Other placental imprints correspond to long terminal repeat (LTR) transposon sequences which serve as alternative promoters for protein-coding genes [121]. These LTR-based imprints are also marked by maternally inherited H3K27me3, but depend on the H3K9 methyltransferase *EHMT1*(G9A) in order to undergo DNA methylation, which likewise occurs upon implantation [114, 122, 123]. Two established examples of organism-wide non-canonical imprints exist in mice [124, 125], but the vast majority of non-canonical imprinting is specific to placenta [114].

A high degree of species specificity is notable in placental imprinting. While many examples of placenta-specific imprinting occur in both primates and mice, in primates the “incomplete reprogramming” mechanism predominates, while in mice, non-canonical imprinting is the primary mechanism for placental imprinting [114, 115, 126]. Furthermore, while a majority of globally imprinted loci in mice are also imprinted in humans [127], almost no placenta-specific imprints are conserved [128]. The placenta is a rapidly evolving organ, existing only in eutherian mammals and showing dramatic morphological variation between different mammals [129]. Divergent sets of imprints may facilitate this rapid evolution.

### A note on primitive endoderm

Of the five cell lineages that undergo global de novo methylation (epiblast, trophoblast, primitive endoderm, sperm, oocyte), primitive endoderm and the tissues it forms receive by far the least scientific attention despite being critical for mammalian development. The primitive endoderm gives rise to the parietal and visceral endoderm, which give rise to the parietal and visceral yolk sac endoderm, respectively [130]. The visceral endoderm guides embryonic patterning and the yolk sacs perform critical nutrient and gas exchange before the placenta has developed [131, 132]. In mice, it also has been established that some visceral endoderm cells are incorporated into gut tube and thus become part of the embryo proper [133–135].

Interestingly, the primitive endoderm may contain a rather placenta-like methylome. Murine extraembryonic endoderm (XEN) cells derived from primitive endoderm have a methylome globally more similar to that of mTSCs than epiblast-derived stem cells [136]. During the peri-implantation period, the primitive endoderm acquires even less methylation than the trophoblast and shows similar features, such as PMDs [31, 32]. Likewise, non-canonical placental imprints show parent-of-origin expression patterns in murine visceral endoderm [121].

While the trophoblast and primitive endoderm are superficially similar insofar as they are both extraembryonic, in early development the primitive endoderm forms from the inner cell mass along with the epiblast, and at the transcriptional level the primitive endoderm is initially far more similar to the epiblast than trophoblast [137]. The primitive endoderm may thus acquire a trophoblast-like methylome by a developmental trajectory very unlike that of trophoblast.

## The function of DNA methylation in placenta

At the cellular level, DNA methylation does not appear to be altogether essential for trophoblasts. *Dnmt1*^*−/−*^* Dnmt3a*^*−/−*^* Dnmt3b*^*−/−*^ murine trophoblast stem cells (mTSCs) can survive in the complete absence of DNA methylation, though they show dysregulation of imprinted genes and upregulation of some differentiation markers [138]. Sakaue and colleagues also conducted an experiment in which they transferred nuclei from *Dnmt1*^*−/−*^* 3a*^*−/−*^* 3b*^*−/−*^ embryonic stem cells to enucleated oocytes, allowed the oocytes to develop to morula stage, aggregated with WT morulas and implanted. The cells from the *Dnmt1*^*−/−*^* 3a*^*−/−*^* 3b*^*−/−*^ morulas were capable of contributing to the placental but not somatic lineage in the resulting mouse, although the degree of contribution was much lower than what is observed for WT morulas [138]. Human TSCs generated via transdifferentiation of naïve human embryonic stem cells have essentially no correct imprinting, but can still grow and differentiate normally in vitro [139].

In both mice and humans, however, DNA methylation is clearly essential for placental organogenesis. Female *Dnmt3l*^*−/−*^ mice, which have heavily hypomethylated oocytes [118], give rise to progeny that fails to progress beyond E10.5 and shows extensive placental malformation [140], indicating that imprints are essential for normal placental development. Loss of maternal *Dnmt3a* and *3b* likewise results in defects in trophoblast adhesion, partially attributable to hypomethylation and overexpression of the imprinted gene *Scml2* [141].

In humans, “androgenetic pregnancies” can occur in which all DNA is of male origin [142]. Such pregnancies may arise if fertilization causes exclusion of maternal DNA or if the starting oocyte is anucleate to begin with [143]. Moles may form via fertilization by two sperm, or via fertilization by a single sperm which undergoes endoreduplication. Either way, the resulting conceptus is genetically normal, diploid, but with a uniformly paternal imprinting pattern. The conceptus becomes a “hydatidiform mole”, which features a lack of embryonic tissue combined with disorganized, hypertrophic trophoblast villi [143]. Hydatidiform moles in turn are 2,000–4,000 times more likely to give rise to placental cancers called choriocarcinomas than normal pregnancies [144]. Androgenetic murine embryos do not give rise to moles but, by mid-embryogenesis, feature dramatically impaired embryonic growth but a normal of amount of trophoblast tissue [145].

Takehashi and colleagues derived hTSCs from hydatidiform moles [146]. The placentally imprinted cell cycle regulator *CDKN1C*(p57KIP) was expressed at far lower levels in molar hTSCs, consistent with maternal expression during normal development. Molar hTSCs, or regular hTSCs in which *CDKN1C* was ablated with CRISPR, failed to show contact inhibition in vitro, potentially explaining why multiple layers of cytotrophoblasts are observed in hydatidiform mole villi and potentially explaining why moles are prone to give rise to choriocarcinoma.

## Conclusions

Having described what is known about the pattern of the placental epigenome and the mechanisms of its formation, there is one major question left. How is the placenta’s distinctive epigenome relevant to its function?

As discussed above, biological roles have been clearly established for a number of placenta-specific imprints [141, 146]. It is easy to theorize that placental CpG island methylation occurs to ensure silencing of somatic genes in placental lineage. This is uncertain though, and it is worth nothing that most CpG islands specifically methylated in placenta are only partially methylated (e.g. Figure 4) and yet show stable silencing.

As for the other distinctive aspects of the placental epigenome, low global 5mC and PMDs, their importance is as yet unknown. Presumably any feature conserved across one hundred million years of evolution must be biologically important, but for now, their significance remains a mystery.

## Data Availability

Not relevant.
